# *Lactobacillus acidophilus* and *Bifidobacterium bifidum* stored at ambient temperature are effective in the treatment of acute diarrhoea

**DOI:** 10.1179/146532810X12858955921159

**Published:** 2010-12

**Authors:** S. Rerksuppaphol, L. Rerksuppaphol

**Affiliations:** Departments of Paediatrics; * Preventive Medicine, Faculty of Medicine, Srinakharinwirot University, Thailand

**Keywords:** diarrhoea, children, probiotics, double-blind trial, infloran

## Abstract

**Introduction:**

Probiotics have demonstrated potential to reduce duration of diarrhoea and frequency of watery stools. Probiotics such as *Lactobacillus acidophilus* and *Bifidobacterium bifidum* (Infloran®) are usually maintained at a storage temperature of 4°C which is generally not feasible in tropical or sub-tropical countries.

**Aim:**

The efficacy of Infloran® for treatment of acute diarrhoea when stored at 28–32°C (room temperature) was evaluated.

**Methods:**

This was a double-blind, randomised study of infants and children aged 2 months to 7 years with acute diarrhoea. Patients were randomly assigned to receive Infloran® stored at 4°C, at room temperature, or to a placebo group. Duration of diarrhoea was a primary outcome, while the number of stools, hospital stay and requirement for rehydration fluid were secondary outcomes.

**Results:**

Probiotics shortened duration of diarrhoea (34.1 and 34.8 hrs when stored either at 4°C or at room temperature, respectively, and 58 hrs with placebo, *p*<0.01) and reduced the number of stools (7.3 and 8 *vs* 15.9 with placebo, *p*<0.01).

**Conclusion:**

Administration of probiotics is beneficial as additional treatment of acute diarrhoea and efficacy is not affected by storage temperature.

## Introduction

Diarrhoea is a frequent cause of morbidity and mortality during infancy and childhood, especially in developing countries.^1^ The World Health Organization advise fluid and electrolytes replacement as the therapy of choice for treatment of dehydration.^2^ However, although the mortality rate is decreased by rehydration,^3^ the duration and number of diarrhoea episodes are not affected.^4^ Probiotics are micro-organisms naturally present in the human digestive tract which maintain a correct balance of intestinal flora.^5^ There is evidence that administration of probiotics as an adjuvant is successful in the management of acute diarrhoea.^6^ However, the majority of studies have been conducted in industrialised countries where nutritional status, drug availability and diarrhoea aetiology are different from those in low-income countries.^7^ Furthermore, each probiotic strain has a different level of efficacy as diarrhoea-adjuvant treatment^8^ and the the type of preparation, killed or lived bacteria, may result in heterogeneous results.^7^,^9^–^11^

In this study we used Infloran® which is comprised of lyophilised live *Lactobacillus acidophilus* and *Bifidobacterium bifidum*. Stability studies indicate that storage at a temperature of 4°C in a normal refrigerator guarantees complete maintenance of live micro-organisms for 24 months, while storage at 22°C (room temperature) decreases the stability time to 4 weeks (described in drug datasheet). In Thailand, however, preserving probiotics in a refrigerator is not generally feasible for all patients and room temperature ranges from 28°C to 32°C. It is reported that at 37°C Infloran® is stable for only 2 weeks and that the amount of live micro-organisms halves during that time. We hypothesise that even if the number of live organisms is reduced by storage at high temperature, killed bacteria may conserve their therapeutic properties. This study examined duration of diarrhoea and decrease in the number of abnormal stools as primary and secondary outcomes, respectively, after administration of refrigerated Infloran® and Infloran® kept at room temperature.

## Subjects and Methods

### Inclusion criteria

A double-blinded, randomised control trial was conducted between January and October 2008 in children aged between 2 months and 7 years admitted to the paediatric unit of Srinakharinwirot University Hospital with a diagnosis of acute diarrhoea. Decisions regarding admission and general management were made, respectively, by the emergency and attending physicians. Children who had passed abnormal watery and/or mucous stools more than three times within the previous 24 hours with duration of diarrhoea for more than 72 hours were eligible for enrolment. Patients with evidence of systemic infections, neurological disturbances, a history of convulsions or conditions such as chronic immunodeficient gastro-intestinal conditions or severe dehydration were excluded.^12^ Children who had received treatment with probiotics and medications which interfere with intestinal motility during the present illness were also excluded. Informed consent was obtained and the study was approved by the ethics committee of the Faculty of Medicine of Srinakharinwirot University.

### Demographic analysis

After enrolment, demographic characteristics and clinical history were recorded. Weight to the nearest 100 g and length/height to the nearest mm were measured. Clinical evidence of dehydration was documented in accordance with WHO guidelines.^12^

### Investigations

Serum sodium, potassium, bicarbonate, blood urea nitrogen, creatinine and complete blood counts were measured. Urinalysis and microscopic faecal examination were routinely performed on admission. Faecal examination for rotavirus by immunochromatography assay (Rota-strip, Coris Bioconcept, Belgium) was undertaken.

### Experimental protocol

After obtaining written consent from care-givers, children were randomised by a computerised programme into one of three groups: to receive live *L. acidophilus* (minimum of 10^9^/capsule) and *B. bifidum* (minimum of 10^9^/capsule) (Infloran®, Berna, Switzerland) stored in a refrigerator (average 4°C); to receive Infloran® stored at room temperature (average 28–32°C) for 1 month before administration; or to receive a placebo stored in a refrigerator. The placebo was a capsule of powdered oral rehydration solution (ORS), identical in colour and size to the Infloran®. Capsules were opened and the powder inside was dissolved in milk or some other fluid before administration. Both patients and attending physicians were blinded to which children were receiving which medication. Children in each group received the assigned medication three times daily until the end of the diarrhoea episode and up to a maximum of 5 days. The end of a diarrhoea episode was defined as the first of two consecutive semi-formed stools or the last stool followed by 12 hours without passing stool. Duration of diarrhoea was a primary outcome and the number of abnormal watery or mucous stools passed, duration of hospital stay and requirement for rehydration fluid were considered secondary outcomes. Adverse events were recorded by interviewing parents or guardians.

### Statistical analysis

Normal distribution of data was tested using the Kolmogorov–Smirnov test. Normally distributed data are presented as mean and standard deviation (SD), and non-normally distributed data are presented as median and interquartile range (IQR). The Pearson *χ*^2^ or Fisher exact tests were used to compare proportions between the groups. Continuous variables were compared by analysis of variance. The least square difference (LSD) method was employed for *post hoc* comparison. Statistical analysis was performed using SPSS 11.0 software package. A *p*-value of <0.05 was considered statistically significant.

## Results

From January to October 2008, 102 potentially eligible patients were approached to enrol in the study. Of these, 67 children accepted and were randomly assigned to the treatment groups: 23 received probiotics stored in a refrigerator, 22 received probiotics stored at room temperature and 22 received a placebo. Two children given probiotics stored at room temperature and a child in the placebo group were withdrawn from the study before receiving medication because of their parents’ concerns. However, the data of all 67 children were included in the intent-to-treat analysis of primary outcome. The data of one child in the placebo group were not analysed for secondary outcomes owing to a later diagnosis of lymphoma.

### Baseline characteristics

Sixty-three per cent of enrolled children were boys with a mean age (range) of 1.9 years (2 months to 7 years). Demographic characteristics, clinical history and biochemical parameters are presented in Table 1. There were no significant differences among the groups in mean age, gender, weight, height, baseline haematological or biochemical parameters. Fifty (74.6%) patients had no or mild dehydration and 35 (52.2%) had been seen by a physician before admission. Twenty-one (31.3%) were infected by rotavirus with no significant difference among the groups. Stool examination was positive for white blood cells in 35 (52.2%) children. The mean number of abnormal stools during admission and duration of diarrhoea before admission were six (range 1–20) and 36 hours (range 1–72), respectively, and these figures were similar among the groups.

**TABLE 1:** Demographic characteristics, clinical history and baseline chemical pathology in the three groups

	Probiotics at 4°C (*n* = 23)	Probiotics at room temperature (*n* = 22)	Placebo (*n* = 22)
Age* (y)	1.0 (2.5)	1.3 (1.6)	1.0 (3.0)
Male, *n* (%)	13 (56.5)	15 (68.2)	14 (63.6)
Weight* (kg)	9.0 (7.6)	10.7 (5.4)	10.2 (8.0)
Length/height* (cm)	77.0 (32.0)	81.0 (24.0)	78.5 (38.9)
Hydration status, *n* (%)			
Mild or no dehydration	18 (78.3)	15 (68.2)	17 (72.3)
Moderate dehydration	5 (21.3)	7 (31.8)	5 (22.7)
No. of diarrhoeal stools in previous 24 hours*	5 (4)	5 (3)	7 (5)
Duration of diarrhoea* (hrs)	24 (66)	24 (55)	24 (6)
Vomiting, *n* (%)	3 (13.0)	7 (31.8)	5 (22.7)
Fever >38.5°C, *n* (%)	7 (30.4)	6 (27.3)	9 (40.9)
Previous visit to a physician, *n* (%)	13 (56.5)	10 (45.5)	12 (54.5)
Sodium (mmol/L), mean (SD)	135 (4)	135 (2)	135 (2)
Potassium (mmol/L), mean (SD)	4.2 (0.6)	3.9 (0.5)	4.1 (0.5)
Bicarbonate (mmol/L), mean (SD)	18 (4)	18 (4)	19 (4)
Urea nitrogen (mmol/L)	4.9 (1.8)	4.4 (2.5)	3.8 (1.5)
Creatinine* (μmol/L)	44.2 (17.7)	44.2 (19.9)	44.2 (8.8)
Urine specific gravity*	1.015 (0.005)	1.018 (0.015)	1.015 (0.010)
Haemoglobin (g/dl), mean (SD)	11.9 (10.8)	11.7 (10)	120.0 (14.5)
White blood cell count* (×10^9^/L)	9.8 (6.3)	10.1 (5.2)	12.0 (6.8)
% Neutrophils*	60.0 (36.3)	52.6 (38.5)	53.4 (35.1)
Presence of WBCs in stool, *n* (%)	12 (52.2)	10 (45.5)	13 (59.1)
Presence of rotavirus, *n* (%)	7 (30.4)	9 (40.9)	5 (22.7)
Treatment with antibiotics after admission, *n* (%)	7 (30.4)	4 (18.2)	8 (36.4)

* Median (interquartile range).

### Outcome measurement

After enrolment, diarrhoea lasted more than 48 hours in half of the patients in the placebo group and the median number of abnormal stools was 12. In particular, probiotics shortened duration of diarrhoea (34.1 and 34.8 hrs when stored either at 4°C or at room temperature, respectively, and 58 hrs with placebo, *p*<0.01) and reduced the number of stools (7.3 and 8 *vs* 15.9 with placebo, *p*<0.01). Regardless of storage, children who received probiotics showed a statistically significant decrease in duration of diarrhoea compared with the placebo group (*p*<0.01) (Table 2). In approximately half (21/45) of the children who received probiotics, diarrhoea ceased within 24 hours and only one-fifth (9/45) had diarrhoea for more than 48 hours. Duration of diarrhoea after receiving probiotics was not significantly different between the two probiotics groups. The secondary outcomes, in particular the total volume of fluid therapy and duration of hospitalisation, were not significantly different between the groups, whereas the number of abnormal stools was significantly different between the probiotics groups and placebo group, but not between the two probiotics groups. No adverse events were reported during the study period.

**TABLE 2:** Outcome measurements

	Probiotics at 4°C (*n* = 23)	Probiotics at room temperature (*n* = 22)	Placebo (*n* = 22)	*p*-value
Duration of diarrhoea (hrs)	28.0 (32.0)*	26.5 (39.0)*	51.5 (44.0)	<0.01
Diarrhoea <24 hrs, n (%)	10 (43.5)*	11 (50.0)*	3 (13.6)	0.03
24–48 hrs	9 (39.1)	6 (27.3)	8 (36.4)	
>48 hrs	4 (17.4)	5 (22.7)	11 (50.0)	
No. of stools per study period	4 (17)*	4.5 (13)*	12 (15)	<0.01
Hospital stay (hrs)	46.0 (42.0)	47.0 (43.0)	64.0 (48.0)	0.23
Total fluid therapy (ml/kg/hr)	6.3 (3.6)	5.4 (2.7)	4.6 (2.3)	0.35

Results are presented as median (interquartile range); * significant difference *vs* placebo (*p*<0.05).

## Discussion

This study demonstrates that probiotics are of benefit to children with acute diarrhoea. Several reports have shown that, in infants and children, probiotics significantly reduce the duration of diarrhoea by more than 3 days.^13^ In particular, major benefits are achieved when there is infection with rotavirus.^13^ However, in developing countries, diarrhoea is often caused by agents other than rotaviruses and infections are often heterogeneous.^14^,^15^ Available data on the benefits of probiotics in acute diarrhoea in low-income countries have demonstrated variable outcomes. Treatment with *Lactobacillus casei* has not shown any benefit in children aged from 3 to 36 months with acute, watery diarrhoea, but lactose malabsorption might have affected these results.^9^ Furthermore, tyndalised (heat-killed) *Lactobacillus acidophilus* has not demonstrated efficacy compared with placebo in acute diarrhoea.^7^ In contrast, *Lactobacillus LB* was found to be an effective and safe treatment for children with diarrhoea for more than 24 hours.^10^ Severity of diarrhoea may interfere with probiotic activity. Suggested inclusion criteria for studying the clinical use of probiotics are diarrhoea duration <7 days and more than three stools in the previous 24 hours.^13^ The present study enrolled children with mainly mild diarrhoea. We included some common parameters important in the management of gastro-intestinal disorders such as degree of dehydration, number of diarrhoeal stools before admission, high-grade fever, absence of hyponatraemia and acidosis (bicarbonate <15 mmol/L) (Table 1).

Whether killed or live microbes are used might determine efficacy as the action of probiotics as biological modifiers is different in the case of live or killed micro-organisms: live probiotics may influence both intestinal microflora and the immune system, while dead cells are able to reduce inflammation in the gastro-intestinal duct.^16^ In adults, lyophilised, heat-killed *L. acidophilus* improves clinical symptoms of chronic diarrhoea in comparison to living lactobacilli.^17^ In infants, heat-killed *L. acidophilus* has a positive effect in the management of non-rotavirus diarrhoea.^18^

Viable Infloran® has already shown efficacy for treating acute diarrhoea in infants in Thailand.^19^ This study demonstrates that, in a warm developing country, probiotics are effective even if stored at room temperature (range 28–32°C). Further studies on the stability and viability of Infloran® will be able to clarify how long probiotics can be stored at room temperature without losing their therapeutic properties for the treatment of diarrhoea.
